# In Field Detection of Downy Mildew Symptoms with Proximal Colour Imaging

**DOI:** 10.3390/s20164380

**Published:** 2020-08-05

**Authors:** Florent Abdelghafour, Barna Keresztes, Christian Germain, Jean-Pierre Da Costa

**Affiliations:** 1 ITAP, Univ. Montpellier, INRAE, Institut Agro—SupAgro, F-34196 Montpellier, France; 2 Univ. Bordeaux, IMS UMR 5218, F-33405 Talence, France; barna.keresztes@ims-bordeaux.fr (B.K.); christian.germain@ims-bordeaux.fr (C.G.); jean-pierre.dacosta@ims-bordeaux.fr (J.-P.D.C.); 3 Centre National de la Recherche Scientifique, IMS UMR 5218, F-33405 Talence, France

**Keywords:** proximal sensing, downy mildew, parametric classification, structure tensor, seed growth segmentation

## Abstract

This paper proposes to study the potentialities of on-board colour imaging for the in-field detection of a textbook case disease: the grapevine downy mildew. It introduces an algorithmic strategy for the detection of various forms of foliar symptoms on proximal high-resolution images. The proposed strategy is based on structure–colour representations and probabilistic models of grapevine tissues. It operates in three steps: (i) Formulating descriptors to extract the characteristic and discriminating properties of each class. They combine the Local Structure Tensors (LST) with colorimetric statistics calculated in pixel’s neighbourhood. (ii) Modelling the statistical distributions of these descriptors in each class. To account for the specific nature of LSTs, the descriptors are mapped in the Log-Euclidean space. In this space, the classes of interest can be modelled with mixtures of multivariate Gaussian distributions. (iii) Assigning each pixel to one of the classes according to its suitability to their models. The decision method is based on a “seed growth segmentation” process. This step exploits statistical criteria derived from the probabilistic model. The resulting processing chain reliably detects downy mildew symptoms and estimates the area of the affected tissues. A leave-one-out cross-validation is conducted on a dataset constituted of a hundred independent images of grapevines affected only by downy mildew and/or abiotic stresses. The proposed method achieves an extensive and accurate recovery of foliar symptoms, with on average, a 83% pixel-wise precision and a 76% pixel-wise recall.

## 1. Introduction

Epidemiological surveillance is a crucial issue related to problems regarding food safety and security, public health and environmental protection. These problems are especially concern viticulture, where the use of phytosanitary products can be intensive, the staff exposition is frequent and the vicinity between residents and vineyards is a cause for concern or even conflicts.

Currently, crop protection practices in vineyards rely mostly on preventive chemical controls. Spraying strategies are usually scheduled according to climatic risks and to the health history of the vineyard [1]. Over the last decade, several alternative spraying strategies have been developed to improve the inputs efficiency. These strategies consider local information regarding the vineyard health status [2]. However, the assessment of risks and health scores (such as the frequency and severity of the disease) still requires the mobilisation of experts scouting vineyards for symptoms. This task is inherently time-consuming and labour-intensive and can only provide a partial and sparse picture of the vineyard’s health [3]. In this context, image processing has been proven one of the most promising automated and non-intrusive techniques. With relatively low costs in terms of instrumentation, labour and time duty, it enables observation of the visible symptoms of grapevines at the scale of the plant and for large acreage [4].

In the last decade, numerous studies investigated the use of imaging for the automated detection of phytopathologies, including those affecting the grapevine. This application scope can be divided between methods exploiting hyperspectral /multispectral images and methods exploiting Red Blue Green (RGB) images. The former exploits mainly the physio-chemical information of spectra which express plant pathogen interactions [5], while the latter takes advantage of the geometrical, colorimetric or textural properties of visible symptoms.

Hyperspectral/multispectral imaging proved to be a very efficient tool to detect early symptoms, sometimes even before they become visible, in the case of grapevine diseases such as trunk diseases or flavescence dorée (or yellowing). However, most parts of the promising results found in the literature were obtained only in controlled laboratory conditions. When replicated for in-field conditions, it proved to be very difficult to achieve satisfying accuracies and to avoid confusions between pathologies and even abiotic stresses [6,7,8,9].

As for colour imaging, the most simple processing method is image binarisation, which consists in discriminating healthy tissues from abnormal ones, thanks to colorimetric thresholds or more rarely thanks to basic textural thresholds [10,11]. These approaches show numerous limitations. They are particularly sensitive to acquisitions conditions, such as, lighting, angle and shades, especially in outdoor conditions. They also perform poorly when confounding factors such as discolourations and other diseases are present in the scene. More elaborate methods rely on machine learning and especially deep learning. Machine learning-based methods exploit a wide range of classification algorithms such as Support Vector Machine (SVM), Random Forest or Bayesian classifiers. Usually, these classifiers are supplied with textural features, mainly Haralick indices and colour indices. Such applications deal with a wide variety of both arable and speciality crops [11,12,13,14]. With the recent development of Convolutional Neural Networks (CNNs) and Regional CNNs (RCNNs), disease classification and detection applications became a very prolific research subject [15]. However, according to a recent review by Boulent et al. [16], both deep learning and conventional machine learning applications deal with the classification of images depicting isolated tissues (mainly leaves), focused on the symptoms and acquired in ideal laboratory conditions. So far, very few studies deal with in-field detection of phytopathologies. The difference is that in the latter case, the problem is not only to differentiate healthy tissues from specific symptoms, but to identify symptoms within a complex pattern of entangled tissues, likely presenting numerous abnormalities and confounding factors. In addition, the resolution exploited in these cases is far lower and the acquisition conditions more variable and detrimental for the estimation of models. In addition these methods are data intensive and suffer from a lack of robustness (due to overfitting scenarios) and a lack of operability in farming applications.

Recent studies proposed more operable strategies for the automatic detection of grapevine diseases. The authors of [17] proposed comparing methods based on Scale-Invariant Feature Transform (SIFT) encoding and deep learning strategies for the real-time detection of Esca and Flavescence dorée diseases. The authors of [18] proposed a deep learning training strategy for the detection of downy mildew in real conditions and show the difficulties of building a robust and replicable model and the requirements in accurate annotations of the database. Both papers show that it is often the properties of the disease and the appearance of symptoms that drives the processing strategy

Authors previously presented a prototype approach, or the detection of downy mildew (*Plasmapora vticola*), based on the statistical structure–colour modelling of proximal sensing RGB images [19]. While able to identify a great diversity of complex symptoms within the grapevine canopy, the method resulted in a poor precision due to the substantial number of potential false positive within a single image of a plant. The preliminary observations drawn from this previous work led authors to propose a more in depth and complete processing strategy to ensure greater accuracy in the detection of the disease.

The purpose of this work is to propose methodological tools and an image processing chain. Both are dedicated to the detection of symptoms due to downy mildew (*Plasmopara viticola*) on proximal colour images acquired directly in the vineyard. These images are obtained thanks to an autonomous embedded device, able to operate in farming conditions with high throughput. It shows the potential and benefits of “frugal” artificial vision for farming applications. The advantages of this strategy is that it is operable “on the go”, i.e., in the same vein as a phytopathology expert that would browse the canopy seeking symptoms. In this case, it is a high-throughput sensor that acquires scenes of vegetation from a farming equipment cruising at a conventional working rate. It is designed to identify small symptoms (<5 cm), which sometimes represent only 12-pixel radius patches on the images, spread in a complex pattern of organs and textures. There is no manual extraction of the affected tissues from the plant nor specific targeting to constitute the database. Downy mildew is an interesting case study because it affects the majority of the world’s vineyards and represents a substantial financial and logistical cost and a major environmental impact. In addition, this pathology presents a wide variety of symptoms with very discrete forms for early occurrences, which is an ideal context to develop versatile algorithms able to adjust to different environments while being trained on moderate databases.

The proposed methodology relies on the parametric modelling of structure–colour features within leaves affected by Downy mildew as well as healthy vine tissues. Based on these models, the detection of symptoms is achieved through a seed growth segmentation. The paper presents a new structure–colour representation called *TC-LEST* (Tensorial Colour Log-Euclidean Structure Tensor), which is an adaptation of the CELEST representation, previously presented in [20] for the pixel-wise classification of healthy vine organs. In addition, it introduces two statical criteria (“Within” and “Between”), based on Mahalanobis distances between features and models. These criteria enable to determine the affiliation of samples to statistical models. Altogether, these contributions are gathered within a framework dedicated to the application of interest, i.e., the detection of downy mildew symptoms. This strategy is conceived to be a relevant alternative to applications relying on deep learning. The purpose is to produce efficient models with minimal data, resulting in agronomically interpretable indicators.

## 2. Plant Material and Instrumentation

### 2.1. Cultivation Environment

Images were acquired at Le Domaine de la Grande Ferrade, a public experimental facility of INRAE ((the French) National Institute of Agriculture, Food and Environmental Research) in the area of Bordeaux. Images were taken on two 0.3 ha plots planted with the red wine grape variety Merlot Noir. One of the plots is cultivated with integrated crop protection and the other according to organic standards. For both plots, phytosanitary inputs are reduced to 50% of the conventional prescribed dose. The plants were affected only by downy mildew and abiotic stresses. At the end of July 2018, the plots were extensively photographed weekly with examples of healthy vinestocks and examples of vinestocks with early and late symptoms corresponding to phenological stages between BBCH (Biologische Bundesanstalt, Bundessortenamt und CHemische Industrie) 75 (berries pea-sized, bunches hang) and 79 (majority of berries touching) [21].

### 2.2. Instrumentation

**Figure 1:**
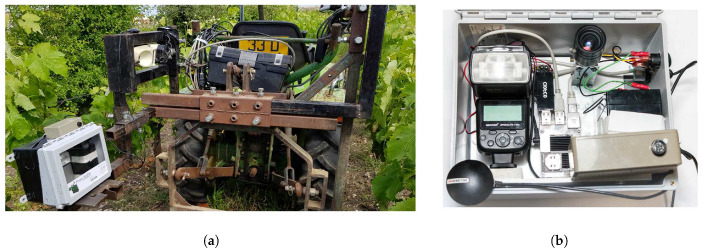
Instrumentation: an autonomous in-field imaging sensor, (**a**) a device embedded on a vine tractor and (**b**) details and elements of the device.

**Figure 2:**
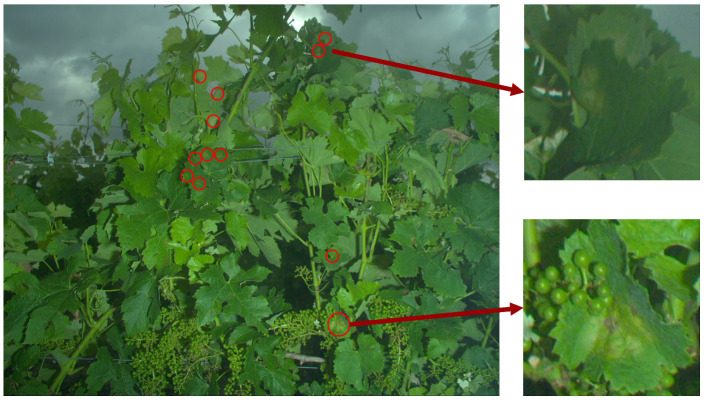
Typical image resulting from the device. The image presents various symptoms of downy mildew circled in red. Two examples of typical “oil spots” are detailed.

The imaging system is composed of a global shutter 5 Mpx industrial Basler Ace (acA2500-14gc GigE) RGB camera with a $55^{{}^{\circ}}$ horizontal field of view lens. To overcome the weather- and time-dependent variations of outdoor illuminations in outdoor environments, the imaging system includes a high-power 58GN xenon flash (Neewer speedlite 750ii) used with a short exposure time (250–300 μs). All the components are powered by a 12 V battery. The device is equipped with an on-board industrial computer that simultaneously controls the shooting of the camera and the trigger of the flash, and stores the acquired image data. The computer is built around a low consumption 4-core ARM chip robust to vibrations and watertight. The device (Figure 1b) is embedded on a vineyard tractor at 70 cm above ground and at 50cm from the target (Figure 1a). At this distance, each image (2592×2048 px) covers a 1.3 m${}^{2}$ area which enables to approximately capture a vinestock and its full canopy at a resolution of 4 px·mm${}^{-1}$. With this resolution, even some early and discrete symptoms are clearly visible. However, at this scale, they are faded within a complex tangle of tissues (see Figure 2). Acquisitions are adapted for the work rate speed in vineyards (3–8 km·h${}^{-1}$), i.e., to ensure one image every meter.

## 3. Image Processing Pipeline

**Figure 3:**
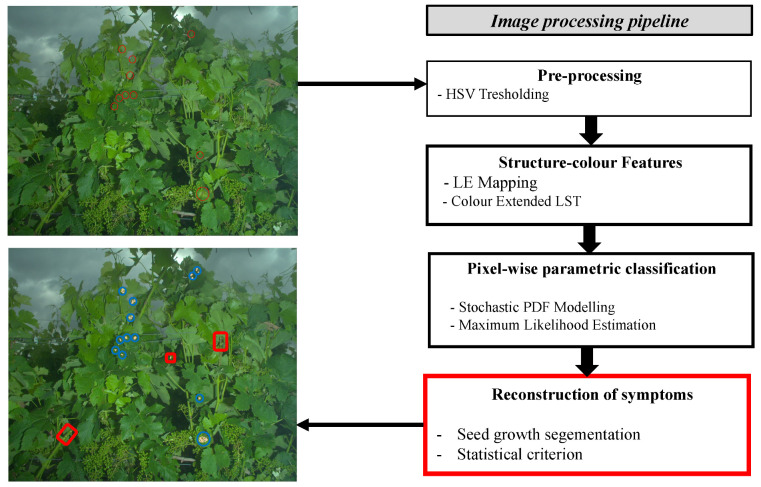
Image processing pipeline for the reconstruction of downy mildew foliar symptoms.

The image processing pipeline is designed to detect the presence of downy mildew symptoms among a wide range of organs and textures (leaves, fruits, stems, necrosis, etc.). The process is primarily based on the statistical modelling of the local structure–colour properties in each of the considered classes. The statistical models enable to determine the likelihoods to the considered classes for each pixel. Then, the likelihoods are evaluated through statistical tests combined with spatial coherence criteria within a seed growth segmentation that determines which pixels could constitute symptoms. The following sub-parts aim at describing the main steps of the proposed processing chain (Figure 3).

**Figure 4:**
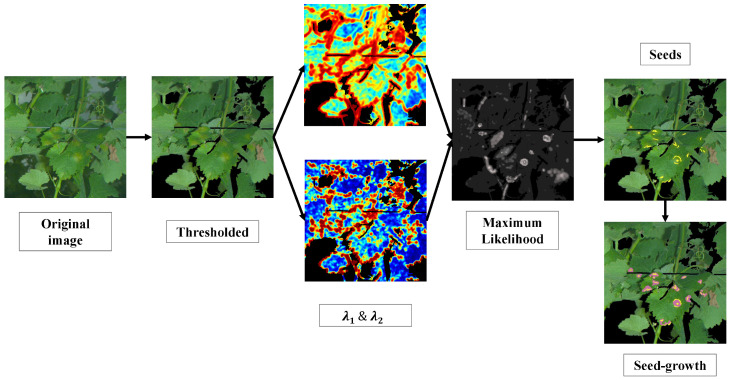
Graphical resume of the processing pipeline.

The first step of the pipeline is the thresholding of images in the Hue Saturation Value (HSV) colour space. The purpose is to discard, on the sole basis of colour, irrelevant pixels that do not constitute plant tissues (such as the sky, poles, wires, etc.). To a lesser extent, it also eliminates overly bright or overly dark pixels. This step is achieved simply by calculating the histogram of hue vales and then applying the Otsu threshold [22]. After this step, the core processes are applied to a limited number of relevant pixels. Images are then processed through several filters to extract features, estimate models and eventually determine iteratively and for each pixel the affiliation to symptoms of mildew. These processes are illustrated with a practical example in Figure 4. When applied to a single 5 Mpx image, the whole detection process requires a moderate computational cost with a unit execution time below 1 s. While the estimation of models from a hundred 5 Mpx images can require several hours with a standard high-frequency CPU, the application itself can be conducted in real-time once the offline modelling phase is achieved.

The remainder of the section will detail the three following major steps; LE mapping, modelling and seed growth algorithm.

### 3.1. Joint Structure–Colour Features

#### 3.1.1. Local Structure Tensor: A Tool to Extract and Represent Textural Information

The LST is a reference tool [23] that extracts geometric information and orientation trends in local patterns within greyscale images. It is commonly defined as the local covariance of gradients [24,25]. The computation of a LST field is a two step process, starting with estimating local gradients in the neighbourhood of every pixel in an image. Given a greyscale image *I* of size [M×N], the gradient image $\vec{\nabla }I$ is estimated as

(1) $$\vec{\nabla }I={\left[g_{x},g_{y}\right]}^{t}={\left[I*\frac{\partial G(x,y)}{\partial x},I*\frac{\partial G(x,y)}{\partial y}\right]}^{t},$$

where *t* denotes the matrix transpose operator; ∗ denotes convolution; and $g_{x}$ and $g_{y}$ represent, respectively, the estimates of the horizontal and vertical derivatives of image *I* obtained by applying Gaussian derivative kernels $\frac{\partial G(x,y)}{\partial x}$ and $\frac{\partial G(x,y)}{\partial y}$. The LST field is then computed by smoothing the outer product $\vec{\nabla }I\vec{\nabla }I^{t}$ with a Gaussian filter $W_{T}$:

(2) $$Y=W_{T}*\vec{\nabla }I\vec{\nabla }I^{t}=W_{T}*\left[\begin{array}{cc} g_{x}\cdot g_{x} & g_{x}\cdot g_{y}\\ g_{x}\cdot g_{y} & g_{y}\cdot g_{y} \end{array}\right].$$

Thus, for every pixel (i,j)∈[1,N]×[1,M] there is a corresponding local structure tensor, Y(i,j) in the form of a 2×2 symmetric matrix:

(3) $$Y\left(i,j\right)=\left[\begin{array}{cc} \sigma_{xx}\left(i,j\right) & \sigma_{xy}\left(i,j\right)\\ \sigma_{xy}\left(i,j\right) & \sigma_{yy}\left(i,j\right) \end{array}\right]$$

#### 3.1.2. Log-Euclidean (LE) Mapping of LST’s

LST’s being covariance matrices, they belong to the Riemannian manifold of Symmetric Positive-Definite (SPD) matrices. The use of standard tools of Euclidean geometry and Gaussian statistics on such variables is not straightforward and shall be carried out by considering the properties of the Riemannian manifold [26]. The mapping of LST’s into the Log-Euclidean (LE) space, as proposed by [26], enables successful image classification in agricultural applications [19,20,27].

The mapping of a tensor Y onto the LE space is achieved by computing its matrix logarithm. Let us consider the eigen decomposition of a LST *Y* as

(4) $$Y=RDR^{-1},$$

where $D=\left[\begin{array}{cc} \lambda_{1} & 0\\ 0 & \lambda_{2} \end{array}\right]$ is the eigenvalues diagonal matrix with $\lambda_{1}$ ≥ $\lambda_{2}$ and $R=\left[\begin{array}{cc} \cos\theta & -\sin\theta \\ \sin\theta & \cos\theta \end{array}\right]$ is the rotation matrix defined by its angle θ. Then,

(5) $$log_{m}\left(Y\right)=R\left[\begin{array}{cc} log(\lambda_{1}) & 0\\ 0 & log(\lambda_{2}) \end{array}\right]R^{-1}.$$

#### 3.1.3. Rotation Invariance

Here, we propose to express a rotation invariant form of Arsigny’s representation in the LE space. Indeed, orientation itself is not a relevant information, as any given tissue, healthy or diseased, should be considered the same regardless of their orientation in the image. As the diagonal matrix of a given tensor provides a unique set of eigenvalues for different possible rotation matrices, it is possible to ensure rotation invariance by retaining only the eigenvalues.

Then, a rotation invariant [20] form of the LE representation $log_{m}\left(Y\right)$ can be easily expressed as

(6) $${\vec{Y}}_{LE}={\left[log\left(\lambda_{1}\right),log\left(\lambda_{2}\right)\right]}^{t}.$$

#### 3.1.4. Describing Grapevine Healthy and Symptomatic Tissues with LSTs

**Figure 5:**
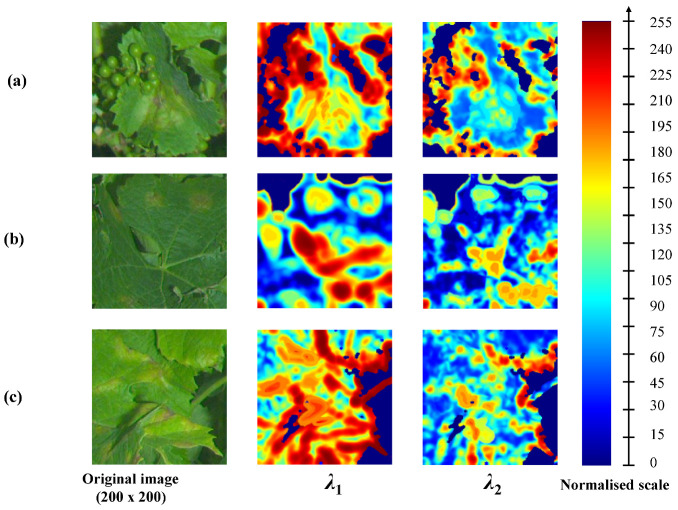
Discriminative properties of LST: separability of visible foliar symptoms of downy mildew on the basis of the $\lambda_{1}$ and $\lambda_{2}$ eigenvalues of the tensor. Eigenvalues are displayed on a normalised 8-bit colour scale. Three “textbook” cases are presented, (**a**) a large circular “oilspot”, (**b**) a “crown of oilspots” and (**c**) irregular symptoms on the edges of the limbus.

Some textural differences between healthy limbus and downy mildew foliar symptoms can be highlighted through features derived from LSTs, computed from the luminance of the greyscale image. Figure 5 illustrates the discriminative potential of LSTs for different facies of the disease. The figure presents the eigenvalues of the LST field mapped into the LE space. Eigenvalues are normalised into an 8-bit scale. Three examples are presented: (a) a large circular advanced oil spot, a corolla of small early oil spots and (c) irregular symptoms on edges. In case (a), it is simple to discern the spot, which medium eigenvalues ([~100, ~120]) differ greatly from smooth limbus presenting low values both for $\lambda_{1}$ and $\lambda_{2}$. In case (b), it is also possible to distinguish some of the symptomatic spots. However, the eigenvalues, especially in the centre of the spots, can be easily mistaken with some leaf edges or some veinlets. In case (c), the interpretation of eigenvalues is more complex due to the irregular shape of symptoms. The inner parts of symptoms present properties and eigenvalues are similar to the previous cases. However, unlike cases (a) and (b), the texture in (c) is not isotropic, thus in the parts adjacent to edges, the structures present dominant orientations and are indistinguishable from healthy leaf edges or limits between organs. Therefore, the structural information alone is not always sufficient to properly discriminate healthy grapevine tissues from pathological ones [19].

### 3.2. Joint Representation of Structure and Colour

Texture and colour are two naturally related properties. It is this relation that enables the human psychovisual system to construct images [28]. Therefore, several methods were developed to extract and represent texture-colour features. In particular, different colour extended LST or LST defined within colour spaces proved to be relevant for image processing applications [29,30,31,32].

Considering the properties of the existing colour LST, and adapting them to the proposed modelling and likelihood based decisions, a novel structure–colour representation is introduced: *TC-LEST* (Tensorial Colour Log-Euclidean Structure Tensor). *TC-LEST* is a refinement of CELEST (Colour Extended Log-Euclidean Structure Tensor), a previous representation proposed in [20]. *TC-LEST* is obtained by mapping LST’s into the Log-Euclidean metric space and then concatenating local colorimetric information. The originality of *TC-LEST* is that the colour components are expressed with as tensor. The result is a low-dimensional vectorial representation describing jointly structure and colour that can be modelled and exploited through common statistical and Bayesian tools.

#### Tensorial Representation of Colour in the HSL Colour Space: *TC-LEST* Representation

An astute method to concatenate colorimetric data to structural information is inspired by the authors of [31]. They proposed to transform the RGB triplet into an Hue, Saturation, Luminance (HSL) triplet before representing it by an ellipse or, equivalently, by a tensor, i.e., a SPD matrix *Z*. The ellipse is constructed so that its orientation φ, eccentricity and magnitude are given respectively by the H, S and L channels. As for the tensor form *Z*, the orientation φ and eigenvalues $\eta_{1}$ and $\eta_{2}$ are deduced from the H, S and L channels as follows,

(7) $$\left.\begin{array}{c} \varphi =H\\ S=1-\frac{\eta_{2}}{\eta_{1}}\\ L=\eta_{1}+\eta_{2} \end{array}\right\}\;\Rightarrow \;\begin{array}{c} H=\varphi \\ \eta_{1}=\frac{L}{2-S}\\ \eta_{2}=\frac{L(1-S)}{2-S} \end{array}$$

The tensorial representation of colours is then expressed as

(8) $$Z=\left[\begin{array}{cc} Z_{00} & Z_{01}\\ Z_{01} & Z_{11} \end{array}\right]=\left[\begin{array}{cc} \cos(\varphi ) & -\sin(\varphi )\\ \sin(\varphi ) & \cos(\varphi ) \end{array}\right]\left[\begin{array}{cc} \eta_{1} & 0\\ 0 & \eta_{2} \end{array}\right]\left[\begin{array}{cc} \cos(\varphi ) & \sin(\varphi )\\ -\sin(\varphi ) & \cos(\varphi ) \end{array}\right]$$

In this form, the computation of dissimilarity measurements and likelihoods with colour components is more relevant and consistent with LST’s statistical models.

Subsequently, it is proposed to process colour with respect to the properties of SPD’s, following three steps. (i) The matrix Z is smoothed by convolution with a Gaussian kernel $Z^{\prime }=W_{C}*Z$. (ii) Then, Z’ is mapped into the LE space similar to LST’s: $Z_{LE}=log_{m}\left(Z^{\prime }\right)$. (iii) The matrix is transformed into a 3-dimensional vector: $\vec{Z}={\left[log\left(Z_{00}^{\prime }\right),log\left(Z_{11}^{\prime }\right),\sqrt{2}log\left(Z_{01}^{\prime }\right)\right]}^{t}$ (consistently with the work in [26]).

Unlike for the structural component, it is senseless to produce rotation invariant colour matrix and it would discard the information contained in φ, i.e., relative to Hue.

Eventually, TC−LEST representation is obtained by concatenating structure and colour into a single 5-dimensional vector:

(9) $${\vec{Y}}_{TC-LEST}=[log{\left(\lambda_{1},log\left(\lambda_{2}\right),log\left(Z_{00}^{\prime }\right),log\left(Z_{11}^{\prime }\right),\sqrt{2}log\left(Z_{01}^{\prime }\right)\right]}^{t}$$

### 3.3. Modelling Structure-Colour Features

Several authors successfully modelled different classes of texture with Gaussian probability density functions that describe the distribution of LSTs within each class. These models have been considered both for the matrix form with Gaussian Riemannian models and for the vectorial form in the Log-Euclidean space and have been applied to the classification of remote-sensed natural textures [20,33,34]. In this article, it is proposed to evaluate such models in the LE space for structure–colour variables derived from proximal sensing images.

Within each of the considered classes, the distribution of colour extended LSTs can be described in the LE space by a multivariate Gaussian function. For a given class, the probability density function is defined by the following equation,

(10) $$p\left(Y|\mu ,\Sigma \right)=\frac{1}{\sqrt{{\left(2\pi \right)}^{D}\left|\Sigma \right|}}exp\left[-\frac{1}{2}{\left(Y-\mu \right)}^{t}\Sigma^{-1}\left(Y-\mu \right)\right],$$

where μ denotes the mean vector of size [D×1], Σ, its [D×D] covariance matrix and |Σ| the corresponding determinant.

By nature, the classes of texture are inherently heterogeneous. Assuming that each class results from the grouping of sub-classes (e.g., leaves = upper limbus + under limbus ), it seems then relevant to consider Gaussian mixtures for stochastic modelling. The probability density function corresponding to a mixture of *K* Gaussian multivariate distributions is defined by the following equation,

(11) $$p\left(Y_{LE}|{\left(\omega_{k},\mu_{k},\Sigma_{k}\right)}_{k=1:K}\right)=\sum_{k=1}^{K}\omega_{k}p\left(Y_{LE}|\mu_{k},\Sigma_{k}\right),$$

where $\omega_{k}$ denotes the weight of the $k^{th}$ Gaussian component of the mixture. $\mu_{k}$ and $\Sigma_{k}$ denote, respectively, the barycentre and the covariance matrix of the $k^{th}$ component.

As the structure and colour joint representations presented above lie in high dimension (5) LE-spaces, observing the adequacy of their distributions to multivariate Gaussian probability is not trivial. A solution is to rely on a remarkable property of the transformation of matrices from the Riemannian manifold to the LE space. Saïd et al. [33] enunciate the following equivalence: if the set of matrices is distributed according to a Gaussian Riemannian function, the distribution of the log-determinants is Gaussian as well and the vectorial representations in the LE space are distributed according to a multivariate Gaussian distribution.

**Figure 6:**
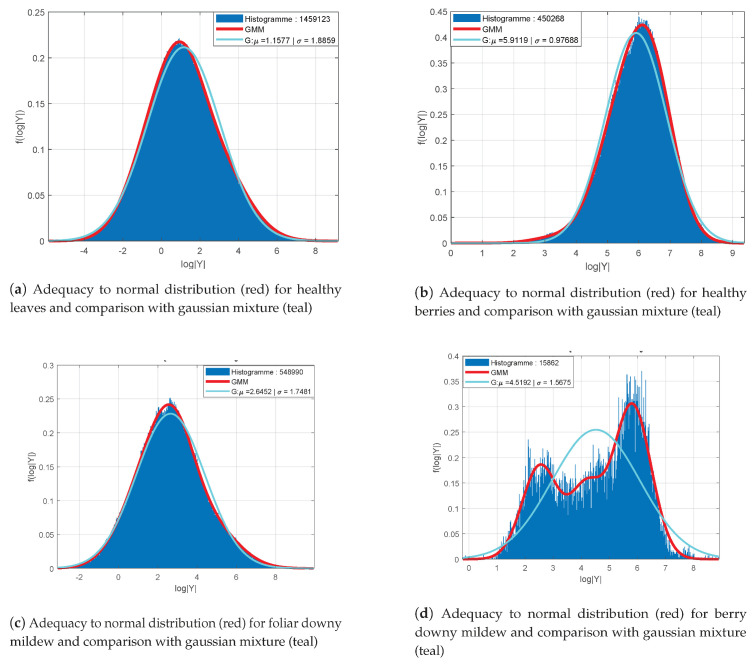
Adequacy of log-determinant distributions (blue) with Gaussian probability functions of same mean and variance (in red) and with Gaussian mixture models or order K = 3 (in teal). Distributions are shown for healthy leaves (**a**), healthy berries (**b**), foliar downy mildew (**c**) and symptoms on berries (**d**).

Figure 6 presents the distribution of log-determinants of LST’s for the classes “healthy leaves” (a), “healthy berries” (b), “foliar symptoms of downy mildew” (c) and “symptoms on berries” (d). Samples are collected from 100 images with a headcount varying between $1.5\times 10^{4}$ and $1.9\times 10^{6}$ depending on the relative abundance of classes within the data base. The histogram of the log-determinants computed from the samples are shown in blue together with the Gaussian distributions of equivalent mean and standard deviation $\left(\hat{\mu },\hat{\sigma }\right)$ represented in red. Gaussian mixtures distributions are shown for the foliar symptoms (c) and the symptoms on berries (d) in teal.

Apart from the class “symptoms on berries” (Figure 6d), all distributions are assimilable to Gaussian probability density functions with their respective empirical mean and variance $\left(\hat{\mu },\hat{\sigma }\right)$ as parameters. However, the distribution for the class “symptoms on berries” can be represented by a mixture of three Gaussian functions (Figure 6d). In addition, the other classes seem to be better represented with mixture models. Indeed, their histograms present some moderate asymmetries and an offset of the distribution’s mode regarding the empirical mean. An example of the fitting improvement provided by mixture models (in red) compared to a single Gaussian model (in teal) is shown for the class “foliar symptoms of downy mildew” in Figure 6c.

*TC-LEST* representations is obtained by concatenating vectorial forms of LSTs in the LE space with colorimetric information. To demonstrate the adequacy of these new variables it is then sufficient to observe their colorimetric components. *TC-LEST*, consists of a vectorial form resulting from the LE transform of a matrices holding the same properties as covariance matrices. Alike LSTs, the distributions of its log-determinants enables then to understand the adequacy to multivariate Gaussian probability functions.

**Figure 7:**
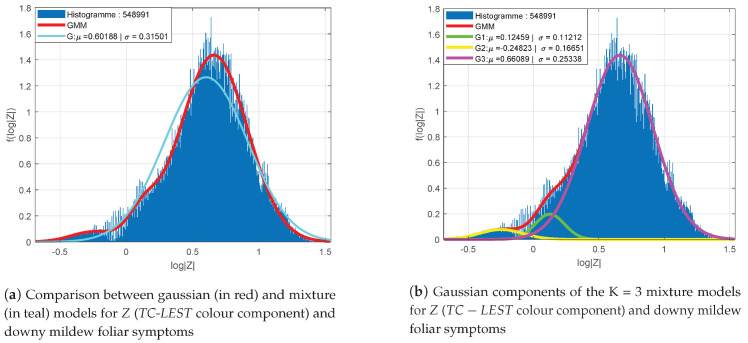
Adequacy of the distribution of log-determinants of *Z* (*TC-LEST* colour component) (red) and comparison with Gaussian mixture (teal) for foliar downy mildew symptoms (**a**). Details of the K = 3 Gaussian mixture (**b**).

Figure 7 presents the distribution of the log-determinants of the colour component of *TC-LEST* for foliar symptoms and their adequacy to a Gaussian probability density function (a) and to a mixture model (b). In this case, the colour component seem to be globally adequate to a Gaussian model, yet with difficulties to represent the mode and the tails. The mixture model is then much more appropriate. In conclusion, the structure–colour representation can altogether be modelled in each class of interest with Gaussian mixture probability density functions.

### 3.4. Seed Growth Segmentation

The use of this segmentation method is motivated by the analogy between “artificial vision” and the human psycho-visual perception system [35]. Indeed, although symptoms of downy mildew present some very distinctive properties at a pixel-wise scale, it is mainly a larger pattern, i.e., a “spot”, which is recognised by an observer. In addition, some elements within symptoms are not easily differentiable from confounding factors. To address this problem, seed growth segmentation considers “spatial coherence”. The purpose is to reconstruct symptoms as continuous connected components constituted of distinctive pixels, arranged coherently in a spatial pattern.

Seed growth [36] is a segmentation method intended to recover connected spaces from a pixel-based classification. This method hinges on two major steps. The first consists in detecting seeds, i.e., the most characteristics pixels within the objects to recover. The seeds are detected by applying restrictive criteria to the decision outcomes of the classification process. The purpose is to maximise the probability that the selected seeds are actually adequate to the model. The second steps consists in aggregating new pixels to the seeds thanks to more permissive and relaxed criteria and under the condition of connexity to the seeds. This second step is intended to propagate confident decisions to recover at best the targeted areas, i.e., foliar symptoms of downy mildew in this case.

In this case, the criteria used are related to the likelihoods between the local structure–colour properties of pixels and the models of the considered classes. These criteria are meant to evaluate Mahalanobis distances [37] between the features describing pixels and the barycentres of the models. These distances convey an information very similar to likelihoods. However, in practice, it is much simpler to evaluate and compare distances than likelihoods. Two criteria of the sort are proposed and combined: a “within” criteria and a “between” criteria that are described in the following.

#### 3.4.1. Within Criteria: Retaining the Most Relevant Pixels of Downy Mildew Symptoms

The within criteria is meant to assess the relevance of a pixel in the class foliar symptoms. It consists in comparing the Mahalanobis distance $d_{mahal}\left(Y,\mu_{mildew}\right)$ between a feature *Y* describing a pixel and the barycentre $\mu_{mildew}$ of the model describing the class of downy mildew foliar symptoms.

Under the hypothesis that a pixel described by *Y* belongs to the mildew class under the normality hypothesis then:

(12) $$Y∼N(\mu_{mildew},\Sigma_{mildew}),$$

$\mu_{mildew}$ et $\Sigma_{mildew}$ being, respectively, the barycentre and the covariance matrix of the mildew model and $d_{mahal}^{2}\left(Y,\mu_{mildew}\right)={\left(Y-\mu_{mildew}\right)}^{t}\Sigma_{mildew}^{-1}\left(Y-\mu_{mildew}\right)$ [37].

Following this hypothesis,

(13) $$d_{mahal}^{2}\left(Y,\mu_{mildew}\right)∼\chi^{2}\left(N\right),$$

where $\chi^{2}\left(N\right)$ is a Chi square distribution with *N* degrees of freedom and *N* is defined by the dimension of the considered descriptors.

A distance threshold $\delta_{\alpha }=\sqrt{\chi_{\alpha }^{2}}$ is then determined so that $p(\chi^{2}<\chi_{\alpha }^{2})=\alpha$, where α∈[0,1] and $\chi_{\alpha }^{2}$ is the α-quantile of the law $\chi^{2}\left(N\right)$. Discarding instances such as $d_{mahal}\left(Y,\mu_{mildew}\right)>\delta_{\alpha }$ is equivalent to retain only a proportion α of the most significant and of the closest instances to the barycentre of the mildew model:

(14) $$d_{mahal}\left(Y,\mu_{mildew}\right)<\delta_{\alpha }\;⟼Y\in c_{mildew}\;\left(\mathrm{class}\mathrm{constituted}\mathrm{of}\mathrm{mildew}\mathrm{symptoms}\right)$$

#### 3.4.2. “Between” Criteria: Discarding Uncertain Pixels

It is possible in some cases that a healthy tissue, yet presenting anomalies, displays properties similar to downy mildew symptoms. In the same way, it is possible that some pixels located at the edges of symptoms resemble, in terms of structure–colour properties, to healthy pixels. In these cases, likelihoods for both healthy and symptomatic classes could be high. Thus, the first criteria cannot prevent such errors.

The “between” criteria consists then in comparing distances between a descriptor and the barycentres of models describing respectively a healthy and a symptomatic class. It enables to determine the pixels for which there is no significant difference in likelihoods between two classes. Considering that symptoms are rare, in such a case where there is a reasonable doubt between two classes, it is more relevant to discard such instances from the mildew class.

This criteria consists then in determining a minimum ratio $R_{min}$, so that if the observed ratio $R_{obs}$ between the squared Mahalanobis distances is lower than this threshold, the instance is discarded from the mildew class:

(15) $$R_{obs}=\frac{d_{mahal}^{2}\left(Y,\mu_{healthy}\right)}{d_{mahal}^{2}\left(Y,\mu_{mildew}\right)}>R_{min}⟼Y\in c_{mildew}.$$

Only values such as $R_{min}>1$ are considered so that the decision criteria always ensure that the maximum likelihood is obtained for the class mildew.

#### 3.4.3. The Seed-Growth Process

To summarise the approach, for all pixels of a given image, the conditions (i) determined by Equation (16) are checked. Pixels that satisfied this equation are considered as seeds. Then, the conditions (ii) determined by Equation (17) are iteratively checked. For each iteration, the pixels satisfying the growth conditions are incorporate to the seeds for the next step. The process eventually stops when no more pixels satisfy the conditions.

(i) Seed selection:

(16) $$\left.\begin{array}{c} d_{mahal}\left(Y,\mu_{mildew}\right)<\delta_{\alpha -seed}\\ R_{obs}>R_{minseed} \end{array}\right\}\begin{array}{c} \;⟹Y\in c_{seed-mildew} \end{array}$$

(ii) Seed growth:

(17) $$\left.\begin{array}{c} d_{mahal}\left(Y,\mu_{mildew}\right)<\delta_{\alpha -growth}\\ R_{obs}>R_{mingrowth}\\ Y\mathrm{connected}\mathrm{to}a\mathrm{seed} \end{array}\right\}\begin{array}{c} \;⟹Y\in c_{mildew} \end{array}$$

with $R_{min-growth}<R_{min-seed}$ and $\alpha_{growth}>\alpha_{seed}$.

## 4. Results & Discussion

### 4.1. Data Set and Validation Protocol

**Figure 8:**
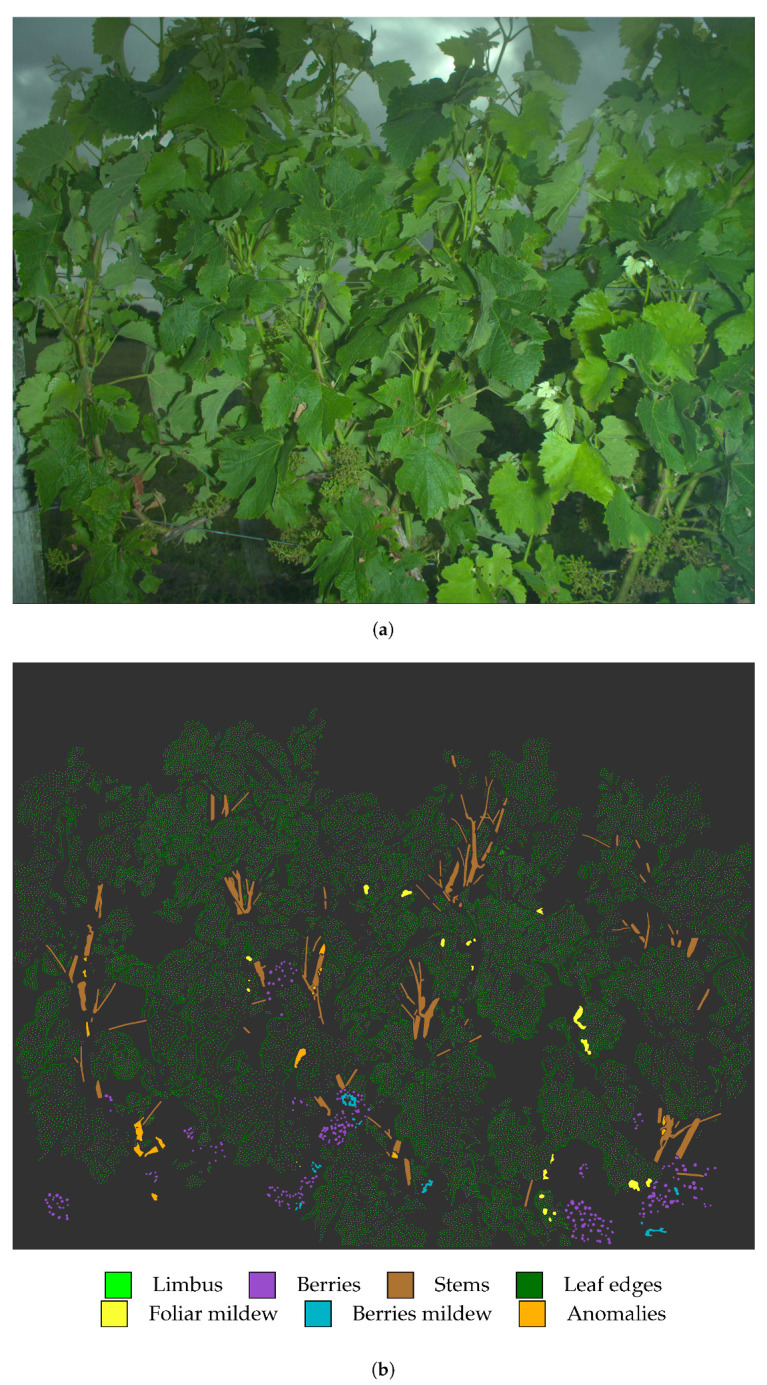
Labelling healthy, symptomatic or abnormal grapevine tissues (**b**) from original (**a**). (**b**) A representative amount of pixel sampled to cover the whole image. Samples are annotated with a colour code corresponding to their class according to photo-interpretation.

Results of downy mildew detection are produced from a dataset containing 100 images acquired mid-June 2018 at the stage “pea-sized berries” (BBCH 75). The dataset contains either healthy plants or plants affected only by downy mildew or abiotic stresses expressed as necrosis or yellowing. The dataset is labelled into seven classes: “Limbus”, “Leaf edges”, “Berries”, “Stems”, “Foliar mildew”, “Berries mildew” and “Anomalies” (i.e., symptoms of abiotic stress). For each image, approximately $5\cdot 10^{4}$ pixels were labelled by photo-interpretation and phytopathological expertise. In total, the database includes $4.7\times 10^{7}$ labelled pixels of which $5.5\times 10^{4}$ belong to the “Foliar mildew” class. Figure 8 presents the example of an image sparsely labelled into into the seven considered classes. In this paper, it is solely the results regarding foliar symptoms which are taken into account. Symptoms on berries are too rare and not well enough modelled so far to be considered in the study. This dataset constitutes both the learning and validation set. Validation is conducted through a leave-one-out cross-validation, i.e., to produce a classification for one of the 100 images, the models are estimated from the 99 remaining images of the set.

### 4.2. Determination of the Seeds

The first step of the reconstruction process (seeds detection) is meant to initialise the detection of symptoms. In practice, it consists in determining for the considered criteria and a couple of thresholds ($\delta_{\alpha },R_{min}$) so that the resulting seeds are both reliable and extensive. The seeds are reliable if they are composed only from pixels within symptoms. They are extensive if there is at least one seed within each symptom to be retrieved.

**Figure 9:**
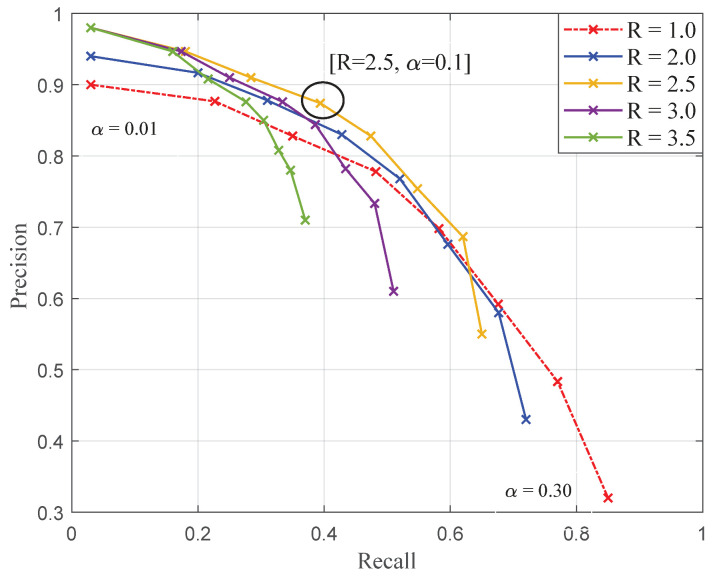
Joint evolution of recall–precision (for mildew pixels) for different combinations of thresholds for the determination of seeds. Each cross represents a value of α∈[0.01,0.30] with low values starting top-left to bottom-right.

Figure 9 presents a set of precision–recall [38] curves describing the classification performances for the classification of downy mildew foliar symptoms depending on different combinations of thresholds ($\delta_{\alpha },R_{min}$). Each of the five curves present the evolution of performances with varying values of $\delta_{\alpha },\alpha \in \left[0.01,0.3\right]$ and for a fixed value of $R_{min}\in \left\{1.0,2.0,2.5,3.0,3.5\right\}$. The red dotted curve corresponding to $R_{min}=1$ constitutes a baseline, i.e., no correction from the maximum likelihood.

The reliability of the seeds is then directly described by the precision. However, extensiveness is only indirectly related to the recall metric. Nonetheless, seeds are all the more likely to be extensive as the recall is high. The purpose is then to determine from the figure a couple of thresholds that satisfy both conditions of seeds, i.e., a trade-off allowing to maximise the precision while ensuring a recall sufficient to initiate each symptom with at least one seed.

Concerning the choice of the “between” criterion threshold, the value $R_{min}=2.5$ stands out. Indeed, this value maximises the precision regardless the value of α, while ensuring the higher recall for any given precision. On another note, the choice concerning the “within” criterion threshold is not trivial. The value for α also has to be determined so that the threshold $\delta_{\alpha }$ maximises the precision while ensuring the extensiveness of seeds. Based on Figure 9, several values for α seem to satisfy condition of reliability of the seeds. Values for α comprised between [0.01:0.15] enable reaching precisions above 80%, but for very variable corresponding recalls, comprised between 3% and 48%. However, pixel-based metrics such as recall cannot fully describe the repartition of seeds within symptoms. For this purpose, it is proposed to evaluate seeds with an additional object oriented metric. Table 1 enumerates for the 100 images of the dataset the number of symptomatic spots which are initialised by at least one seed pixel depending on α values. Thanks to this metric, it can be experimentally determined that the pair of threshold $\left(R_{min}=2.5,\alpha =0.1\right)$ offers the best compromise with reliable seeds (precision = 88%) and is nearly extensive (98% of symptoms initialised).

### 4.3. Seeds Rowth

The determination of seeds only initialises the process of reconstruction of the symptoms. The following expansion around the seeds is a process consisting in aggregating additional pixels to the existing seeds. The purpose is to supplement each seed to form a connected space that represents at best the area covered by the real symptoms. The process is iterative; initially the connected space is composed only by a seed. At each iteration, pixels within the close neighbourhood of seeds that satisfy the resemblance criteria regarding the model for symptoms are added to the pre-existing seed. In this application, the “8-connectivity” neighbourhood is considered for the expansion of seeds. The process stops when there are no more pixels in the vicinity of the connected spaces that satisfy the criteria.

**Figure 10:**
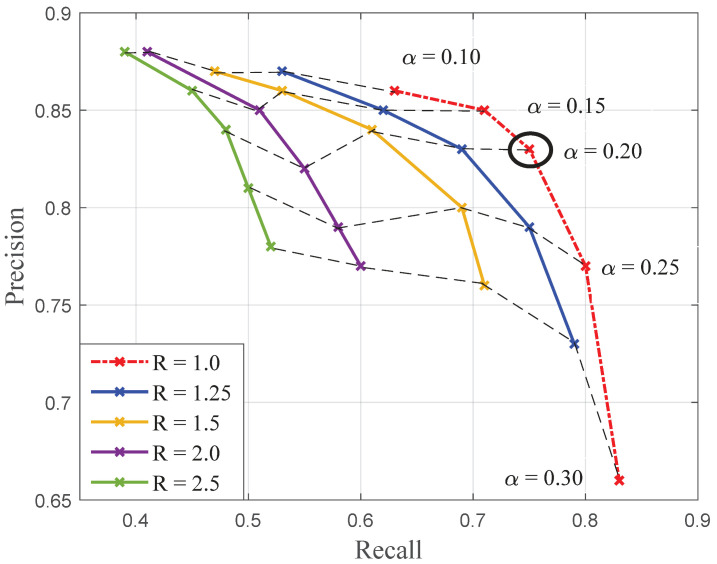
Performances of the reconstruction of downy mildew foliar symptoms for 100 images, depending on the pairs of relaxed thresholds ($R_{min},\alpha$) from seeds determined with $[R_{min}=2.5,\alpha =0.1].$

In this particular case, the “within” and “between” criteria used for the determination of seeds are also used for the expansion of seeds, but with relaxed thresholds. The criteria have to be permissive enough to completely reconstruct the symptoms without downgrading the precision initially obtained for seeds. The new thresholds are then determined experimentally. Figure 10 presents the set of recall–precision curves describing the reconstruction performances corresponding to different pairs of relaxed thresholds ($R_{min},\alpha$) and resulting from seeds obtained with ($R_{min}=2.5,\alpha =0.1$). The “within” criteria is evaluated for relaxed values of α∈[0.1,0.3]. This means that a greater proportion of the population constituting the model for symptoms is considered relevant, i.e., pixels for which the distance $\delta_{\alpha }$ to $\mu_{mildew}$ is greater can be added to the connected spaces. The “between” criteria is evaluated for relaxed values comprised between $R_{min}\in \left[1.0,2.5\right]$.

Figure 10 shows that the reconstruction which is the closest to full completion is obtained for the value $R_{min}=1$. In this case, the “between” criterion does not differ from maximum likelihood with an additional condition of connexity. For values of $R_{min}\leq 1$, the recalls are slightly better but the precisions drop drastically. Concerning the threshold for $\delta_{alpha}$, values of α greater than 0.2 are too permissive. A good compromise can be found for the threshold α=0.2, which results in an 83% precision and a 76% recall.

**Figure 11:**
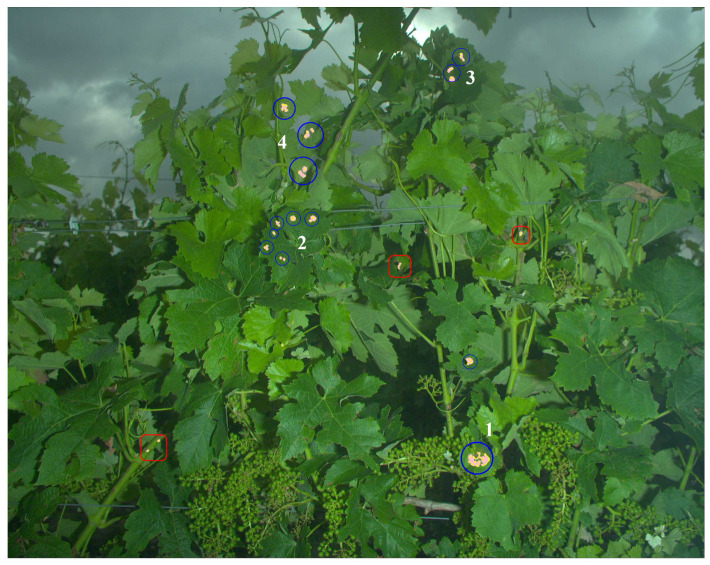
Reconstruction results with the thresholds: (seeds) [$R_{min}=2.5$,α=0.1]⟼ [$R_{min}=1$, α=0.2] (expansion). Blue circles indicate the seeds corresponding to annotated symptoms. Red frames indicate false positives.

Figure 11 shows a representative result for the reconstruction of symptoms resulting from the thresholds ($R_{min}=1,\alpha =0.2$) for the expansion and seeds determined with ($R_{min}=2.5,\alpha =0.1$). The image exhibits 13 symptomatic spots that are all detected (circled in blue). Three errors (false positives framed in red) are produced around some necroses on stems. In terms of area detected for the estimation of sanitary risks, these errors are minor. However, if the objective was to detect the first early symptoms in a plot, these errors could lead to a very different interpretation of the results in terms of epidemiology.

**Figure 12:**
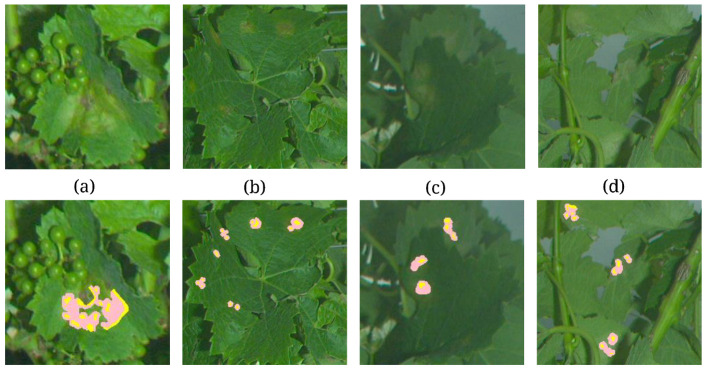
Detailed view of the reconstructed symptoms after growth: from seeds [$R_{min}=2.5$,α=0.1]⟼ [$R_{min}=1$, α=0.2] growth. Seed pixels are represented in yellow, while pixels added during the growth phase are represented in pink. These four examples correspond to the numbered symptoms indicated in Figure 11, (**a**) Large coalesced oilspot, (**b**) crown of necrosing oilspots, (**c**) early adaxial symptoms and (**d**) early abaxial symptoms.

Figure 12 shows the reconstruction of symptoms with finer details. The larger symptoms, or the most severe ones, are generally better retrieved than the smaller and more discrete or faded symptoms. Moreover, if the general area covered by symptoms is rather well estimated and located, the morphology of the symptoms is not necessarily transcribed accurately. However, in the specific case of downy mildew, the shape of the symptoms is not a crucial parameter, as they can coalesce or grow according to very local shades, humidities or wounds. Furthermore, the reconstruction can result in multiple partial detections of a single symptom as a group of close but unconnected symptoms. Indeed, for most symptoms, multiple seeds are detected. In some cases, the expansion phase lead to their coalescence (cf. Figure 12a,b). In other cases the expansion does not fully recover the symptom but solely enables to better estimate the area it covers (cf. Figure 12c,d ). This phenomenon of multiple partial detections could constitute a difficulty for the epidemiological interpretation of the results as the countdown of elementary symptoms is accounted for.

When applied to the whole validation set of a 100 images, the process seems rather efficient. Table 2 shows the variability of the performances within the dataset. For a global estimation within a plot, the results are satisfying. With adequate thresholds, symptoms are retrieved with an 83% precision and a 76% recall. The standard deviation (σ) of these performances is quite modest at the considered scale. However, at the scale of a plant, for some images, the process can be incomplete or lacking in precision. Yet these extreme counter performances do not occur together as described by the minimum $F_{1}$ score equals to 75% ($F_{1}=2$·$\frac{precision\cdot recall}{precision+recall}$).

## 5. Conclusions

The purpose of this work was to evaluate the potential on on-board high-resolution colour imaging for the monitoring of cryptogamic diseases affecting grapevine thanks to a case study: downy mildew (*Plasmopara viticola*). To address the problem, a pixel-wise classification strategy has been validated. First, the relevance of local structure tensors when describing healthy or infected vine tissues was demonstrated. Then, it was proposed to enhance LSTs with colorimetric information. To do so, a novel structure–colour representation (*TC-LEST*) was developed. In addition, it has been shown that thanks to a logarithmic transformation, compact vectorial descriptors were conveniently obtained. These descriptors were proved to be easily modelled within different classes of vine tissues thanks to Gaussian mixture distributions. Finally, these models were used to define statistical criteria that were in turn integrated within a seed growth segmentation process. The proposed strategy was applied to database of a hundred images of vinestocks. Results were evaluated with a leave-one-out cross-validation process in terms of pixel-wise precision and recall. With the best parameters, classification performances reached 83% precision and 76% recall.

These first promising results show that it is possible to discriminate, count and measure foliar symptoms of downy mildew. This contribution should lead to further validation in conditions closer to the agronomical requirements.

## Figures and Tables

**Table 1:** The extensiveness of seeds depending on α values: proportion of symptoms including at least a seed pixel.

α	0.01	0.02	0.05	0.1	0.15	0.20	0.25	0.30
% Symptoms with seeds	49	66	83	98.1	98.5	99.3	99.3	99.3

**Table 2:** Variability of the performances of symptoms reconstruction in the image database: (seeds) [$R_{min}=2.5$,α=0.1]⟼ [$R_{min}=1$, α=0.2] (growth). Performances are computed for the pixel-wise scale.

	Min	Max	Mean	σ
Precision	0.68	0.91	0.83	0.08
Recall	0.48	0.98	0.76	0.16
$F_{1}$-score	0.75	0.89	0.79	0.10

## Abbreviations

The following abbreviations are used in this manuscript.

BBCH	Biologische Bundesanstalt, Bundessortenamt und Chemische Industrie
CELEST	Colour Extended Log-Euclidean Structure Tensor
CNN	Convolutional Neural Network
HSL	Hue Saturation Luminance
LE	Log-Euclidean
LST	Local Structure Tensor
RCNN	Regional Convolutional Neural Network
RGB	Red Blue Green
SIFT	Scale Invariant Feature Transform
SPD	Symmetric Positive Definite
SVM	Support Vector Machine
TC-LEST	Tensorial Colour Log-Euclidean Structure Tensor
